# Processing of high temperature alumina/aluminum titanate ceramic composites from clean sources

**DOI:** 10.1038/s41598-022-09670-3

**Published:** 2022-04-08

**Authors:** Nada H. A. Besisa, Dina H. A. Besisa, Emad M. M. Ewais

**Affiliations:** Refractory & Ceramic Materials Division (RCMD), Central Metallurgical R&D Institute (CMRDI), P.O. Box 87, Helwan, Cairo, 11421 Egypt

**Keywords:** Chemistry, Engineering, Materials science

## Abstract

Producing new technological materials with high performance from clean sources has become a global requirement. Alumina/aluminum titanate (Al_2_O_3_/Al_2_TiO_5_) composites are high-temperature portentous materials used in various advanced applications. In this work, different Al_2_O_3_/Al_2_TiO_5_ composites were obtained with high thermal and mechanical properties for high-temperature applications by a low-cost process. The targeted composites were produced from calcined alumina and, rutile ore extracted from the Egyptian black sands by pressureless sintering at a temperature of 1650 °C/2 h. Rutile was added to alumina with a different content (0–40 wt%) to promote its sinterability and thermo-mechanical response. Evaluation of the produced composites in terms of phase composition, densification, microstructural features, mechanical and thermal properties was investigated. The results indicated that the addition of small amounts of rutile (10 and 20 wt%) succeeded in forming a stable Al_2_O_3_/Al_2_TiO_5_ composite structure. However, higher content of rutile led to the formation of Al_2_TiO_5_ rich matrix composites. Moreover, highly dense composites with harmonic microstructure and enhanced mechanical strength were attained by increasing the rutile content. The composite with only 10 wt% rutile addition gave the highest density of 3.6 g/cm^3^ and the highest cold crushing strength and modulus of rupture values of 488.73 MPa and 106.19 MPa, respectively. Notably, the addition of rutile has a substantial effect on promoting the thermal properties and thermal stability of the obtained composites up to a high temperature of 1400 °C. The present study shows that addition of rutile ore to alumina is one economical way of improving the densification and thermal expansion of Al_2_O_3_ for high temperature applications. Using a clean source such as rutile ore that contains some thermal stabilizers as Fe_2_O_3_, Al_2_O_3_, SiO_2_, ZrO_2_, and MgO instead of pure TiO_2_ has played a noticeable role in improving the reaction sintering and resulting in a highly qualified material. Thus, sintered Al_2_O_3_/Al_2_TiO_5_ composites can be considered as a promising high-temperature material for advanced applications.

## Introduction

Nowadays, with the continuous development of the different sectors of industry, the processing of high-temperature advanced materials has become an urgent requirement. It was established that high temperature materials are those withstanding temperature environments in the range of 500–600 °C^1–4^. So, ceramic and refractory materials were considered as the most promising candidates for high-temperature applications. It was also found that the suitability and sustainability of materials for high temperature applications relied on their high temperature thermal and mechanical performance as well as their production costs. Moreover, from the economic and industrial points of view, one of the most critical issues facing these high-temperature materials is decreasing their cost^5^. Thus, the main challenge is obtaining high-temperature materials with high thermal and mechanical properties with low cost.

One of the most known high-temperature ceramic materials is alumina (Al_2_O_3,_ A). It is a well-known structural ceramic material that can be widely employed in various fields of applications due to its superior properties. Some of these properties are high melting point, chemical inertness, good corrosion resistance, wear resistance, hardness, high insulation, and ease of processing. However, catastrophic failure of alumina occurs in an acute thermal environment owing to large stresses sophisticated over thermal variations. Moreover, despite the high mechanical strength of alumina, its high thermal expansion (α_20–1000 °C_ = 8 * 10^–6^ K^−1^) and thermal conductivity, limit its range for some high-temperature structural applications^6–9^.

Furthermore, aluminum titanate (Al_2_TiO_5_, AT) is a promised high-temperature ceramic material characterized by outstanding thermal shock resistance, high corrosion resistance, and low coefficient of thermal expansion. Hence, it has been considered as a successful candidate in various severe thermal environments, such as thermal processing technology, thermal insulation, refractory, metallurgy, glass and automotive industry, and engine components^8–15^.

Also, AT was used as a ceramic additive to improve the thermal and mechanical properties of some ceramic composites^16^. In addition, some studies have confirmed that the insertion of AT into alumina ceramics enhances their fracture toughness and mechanical properties. This enhancement has resulted from the local residual stresses induced by the large mismatch in the thermal expansion coefficient between A and AT^16–19^. It was also found that the addition of Al_2_TiO_5_ to Al_2_O_3_ composites led to the formation of a new material with better flaw-tolerance properties^17,18^.

Accordingly, combining A and AT into one composite structure will play a vital role in enhancing the thermo- mechanical response of alumina and overcome the noteworthy issues of AT ceramics^20,21^, giving a new tailored material with enhanced characteristics for advanced high-temperature applications. The functional and structural properties of the tailored A/AT ceramics have made them suitable for a vast range of advanced applications, such as exhaust filter components for diesel engines, high-temperature ceramic substrates, thermocouple sheaths, in addition to thermal barrier coating and other applications^6,8,9^.

Limited studies have investigated the preparation and characterization of A/AT ceramic systems^6,8–11,16–19^. In these previous works, A/AT ceramics were obtained by either the solid-state reaction between A and titanium dioxide (TiO_2,_ T) or the direct sintering of A and AT^6,22^. However, the production cost of pure T is relatively high and is a complicated process. To the best of our knowledge, no work has obtained A/AT structures from clean natural sources.

Furthermore, different methods for producing high temperature ceramic materials were reported such as hot pressing, spark plasma sintering, chemical vapor deposition, sol–gel processing, self propagating combustion synthesis and others^16–19^. However, these techniques require complicated processing and highly expensive starting materials. In contrast, pressureless sintering is a very simple and cost effective method. It is the simplest method of shaping material powders and not demands any external force other than the constraints of the mould wall, gravity and atmospheric pressure. It can be provided for sintering of refractory materials as well as oxide ceramics or carbide and nitride materials. So that, from the industrial estimation pressureless sintering method is the most appropriate method to prepare advanced materials with high efficiency and low cost^10,11^.

On the other side, rutile is the most widespread mineral, composed mainly of titanium dioxide. Its refractive index is considered one of the highest indices of all known minerals. It has been used in the manufacturing of refractory and ceramic materials and various industrial applications. It was found that the best economical method for rutile extraction is by recuperating it from weathered deposits in mineral sands^23,24^.

Furthermore, the Egyptian black sands contain several economic minerals. Rutile is considered one of the exceedingly significant minerals in these black sands. Most of the individual economic minerals from the Egyptian black sands, including rutile, can be obtained with marketable grades and accepted recoveries by using different low-cost and simple techniques, such as wet-gravity concentration, and magnetic separation techniques^25^. So, rutile ore extracted from black sand minerals is strongly approved to be the main source of titanium dioxide ceramics.

Once the titanium containing ores (e.g. rutile), have been mined, they have to be covered into pure titanium oxide. One of the main production methods of TiO2 is the chloride process, in which rutile can be used. This process requires large amounts of critical chemicals and remarkable quantities of energy. Apart from waste (solid or liquid) of unreacted minerals or different chlorine compounds, the chloride operation can output gaseous particulates, chlorine and sulfur dioxide emissions^23^. According to variety of problems result from the manufacture of pure titanium dioxide involving high energy cost, consumption of hazardous chemicals, production of plenty amounts of hazardous gases, acidic emissions along with wastes, which could cause considerable harm to the environment. Rutile ore is strongly suggested to be directly used in several industries^24,25^. So that the main target of this work is synthesis of high temperature industrial ceramic materials with optimized thermal and mechanical properties with low cost and environmental friendly method. Consequently, for the first time, advanced high-temperature A/AT ceramic composites were produced from natural clean sources with low-cost processing. Various Al_2_O_3_/Al_2_TiO_5_ composites have been developed with high thermal and mechanical performance for high temperature and advanced applications. The proposed composites were produced from the solid solution reaction of calcined alumina and rutile ore extracted from the Egyptian black at a temperature of 1650 °C/2 h. Rutile was added to alumina with a different content (0–40 wt%) to promote its sinterability and thermo-mechanical response. Besides, using a clean natural source such as rutile ore which already contains some stabilizers as Fe_2_O_3_, Al_2_O_3_, SiO_2_, ZrO_2_, and MgO has played a significant rule in lowering the cost of the preparation process and also in modification of the prepared composites properties. Evaluation of the produced composites in terms of phase composition, densification, microstructural features, mechanical and thermal properties was evaluated and analyzed.

## Experimental procedure

### Materials and processing

In this study, we have considered the preparation of A/AT composites from the solid-state reaction between aluminum and titanium oxides sources. The starting materials used in this work are highly pure (99.4%) calcined alumina with a main particle size of d_50_ = 9.227 µm. It was supplied by SALOX®M-1FG (GKE-MF), Silkem, d.o.o, Tovarniska cesta, Slovenia, Europe. Rutile ore with a main particle size of d_50_ = 2.384 µm, from the beneficiated deposits of the black sands of the north shores, Nuclear Materials Authority of Egypt was used as the source of TiO_2._

**Figure 1:**
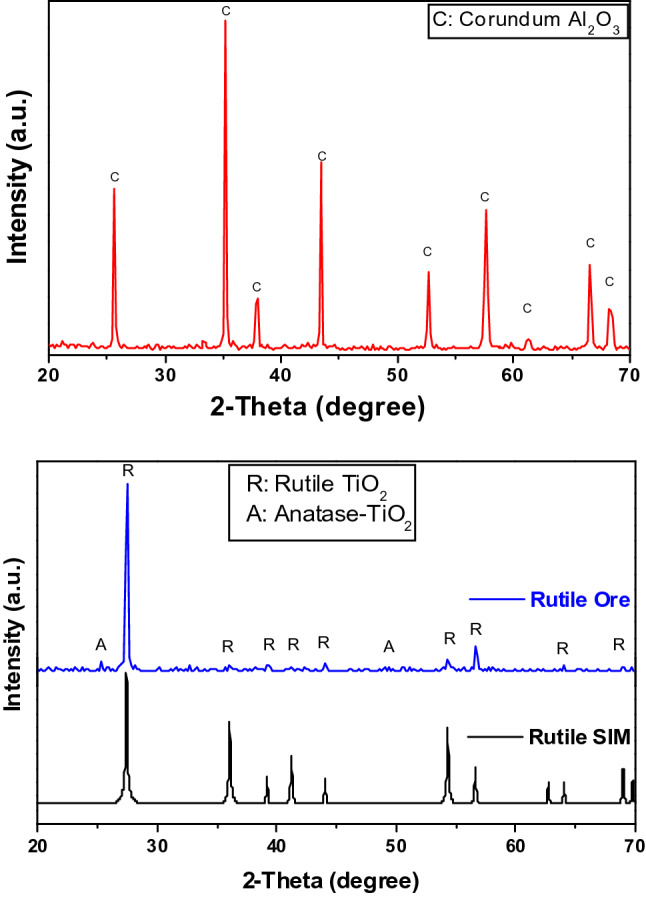
XRD patterns of the starting materials: calcined alumina (**a**) and rutile ore (**b**).

Details of the starting ceramic materials in terms of chemical analysis and phase composition are illustrated in Table 1 and Fig. 1.

**Table 1:** Chemical composition, wt% of the starting materials.

Oxide/element	Al_2_O_3_	TiO_2_	SiO_2_	Fe_2_O_3_	MgO	ZrO_2_	CaO	Na_2_O
Alumina	99.4	–	0.07	0.02	–	–	0.03	0.48
Rutile	4.03	77.95	3.45	8.24	0.39	3.36	–	0.25

Five different ceramic batches of A/AT composites with various rutile ore content (0–40 wt%) were produced by powder metallurgy technology. Samples designation/nomenclature with different percentages of the starting materials of alumina and rutile is illustrated in Table 2. Each composite mixture was homogeneously mixed in ethanol through a planetary ball milling for 2 h. The mixtures were dried and sieved through < 300 μm sieve. Green compacts in cylindrical form with a diameter of 2.5 cm were produced by uniaxial pressing (KPD-30 A, Spain) at 95 MPa. The final composites were produced by pressureless sintering at a temperature of 1650 °C/2 h using an electric furnace (HT 16/17, Nabertherm, Germany). The heating rate of the sintering process was kept constant at 5 °C/min.

**Table 2:** Designation (wt%) of the different A/AT ceramic composites.

Sample	Alumina (wt%)	Rutile (wt%)
0R	100	0
10R	90	10
20R	80	20
30R	70	30
40R	60	40

### Characterization

Phase analysis of the starting and different sintered composites was performed by a Brucker D8-advance X-ray powder diffractometer with Cu Ka radiation (k = 1.5406 Å). The x-ray diffractometer was attached with semi-quantitative measurements to follow up the reaction and phase content in each composite. Panalytical XRF (Model advanced axios, Netherlands) is used to determine the chemical composition of the starting materials.

Densification parameters in terms of bulk density and apparent porosity of the sintered composites were obtained by Archimedes immersion method using ethanol as an aqueous medium, ASTM C 373-72, 1984^26^. Linear shrinkage of the different composites was calculated by determining the diameter of the specimens before and after sintering.

Microstructure properties of the sintered composites were investigated by backscattered electron (BSE) in the field emission scanning electron microscopy (FESEM; QUANTA FEG250, Holland) equipped with an energy dispersive X-ray microanalyzer (EDX).

Linear thermal expansion (LTE) and its coefficient (CTE) were performed by using dilatometry (Linseis Inc., Germany, Model L76/1550) in the temperature range of room temperature up to 1400◦C with a heating and cooling rate of 10 °C/min. The measurement was performed on specimens with a determined diameter of 5 mm and a length of 35 mm. The measurement was also performed on three specimens for each composition of the obtained composites and finally was taken as average values.

Bending strength (Modulus of rupture, MOR) of the specimens was estimated by the three-point bending test using (LFM-L 20 kN, Walter + Baiag, Australian).

Cold crushing strength (CCS) was measured according to ASTM C 1424-04, 2006^27^ using the universal testing machine (SHIMADZU Corporation made in Japan-model UH-F1000KN- Capacity 20-1000KN). The specimen was carefully placed into two load blocks and alignment of the specimen in the load blocks was ensured. The load was slowly applied with a crosshead rate of 1 mm/min. Cold crushing strength (CCS) was calculated according to the following formula^28^:

$${\text{CCS}} = {\text{W}}/{\text{A}}$$

where CCS = Cold crushing strength (N/mm^2^), W = Fracture load (N), A = the cross-section area of the specimen (mm^2^).

## Results and discussion

### Phase composition and densification behavior

**Figure 2:**
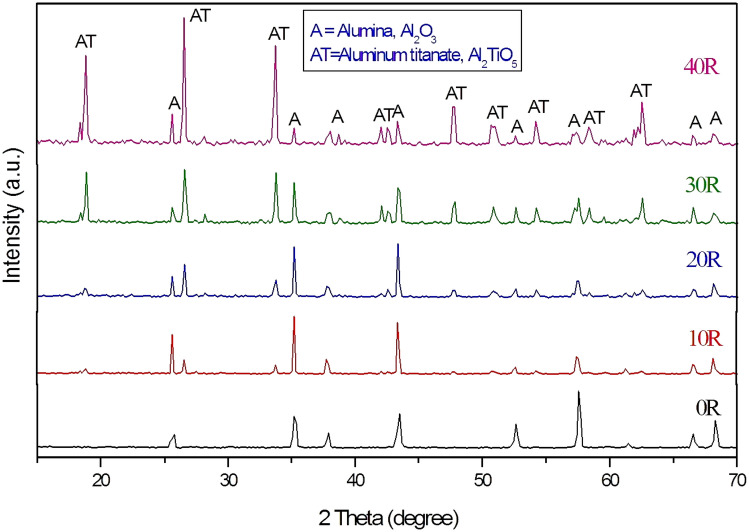
XRD pattern of the sintered A/AT composites with different rutile content (0–40 wt%).

Figure 2 illustrates the XRD patterns of the pressureless sintered A/AT composites at 1650 °C/2 h with different rutile content (0–40 wt%). It was noticed that for composite without rutile addition (0 R), all the identified peaks corresponded to the alumina phase only. However, upon the addition of 10 wt% rutile, aluminum titanate peaks start to appear due to the solid-state reaction formed between alumina and rutile ceramics. Increasing rutile content higher than 20 and 30% led to increasing the intensity of the formed AT peaks and decreasing alumina peaks intensity. For composite with 40% rutile addition (40 R), AT peaks become the dominant phase with a minor appearance of A peaks. So, it can be concluded that the addition of rutile with high content increases the chances of AT formation to a higher extent.

**Figure 3:**
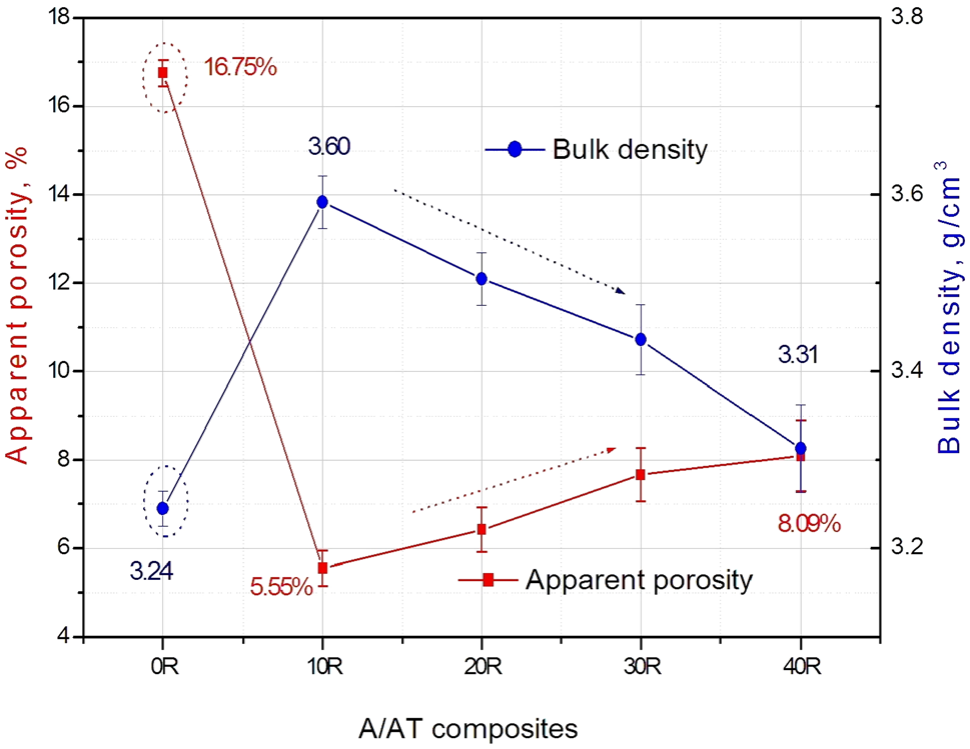
Apparent porosity and bulk density of the sintered A/AT composites with different rutile content (0–40 wt%).

On the other hand, densification parameters in terms of apparent porosity and bulk density of the different sintered composites are measured and calculated as shown in Fig. 3. Composite 0 R without rutile addition has recorded the lowest density of 3.24 g/cm^3^ and the highest porosity of 16.75%. However, upon the addition of 10 wt% rutile to alumina, the density of the sample enhanced and increased to 3.60 g/cm^3^ and the porosity sharply decreased to 5.55%. This considerable enhancement of the densification parameters of A/AT composites upon the addition of small rutile content reflects its effective and positive role in alumina response. This behavior can be attributed to the homogenous diffusion of rutile in the alumina structure and the attained liquid phase reaction. In contrast, this situation is completely changed with increasing rutile content higher than 10 wt%. In another meaning, with increasing rutile content to 20%, density decreased, and porosity increased. Composite with 40% rutile gave density value of 3.31 g/cm^3^ and porosity of about 8%. Yet, these values are still better than those achieved by the 0% rutile composite, Fig. 3. The slight reduction in the densification parameters of A/AT composites with the addition of a higher content of rutile (20–40 wt%) can be attributed to the formation of AT with higher content (as explained in XRD part, Fig. 2). It is well known that it is very difficult to attain a fully dense sintered AT structure^6,10^. Moreover, AT has a lower density than alumina^10,11^. Consequently, increasing the amount of formed AT leads to decreasing the densification of the obtained structure.

**Figure 4:**
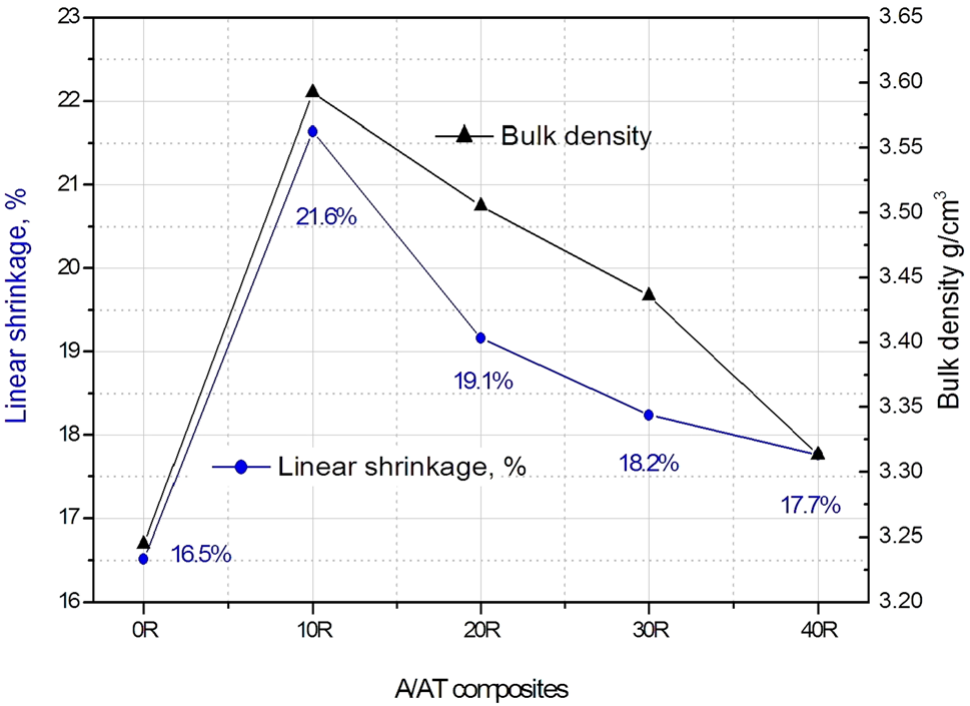
Linear shrinkage, % and bulk density of the sintered A/AT composites with different rutile content (0–40 wt%).

Shrinkage is a characteristic way of determining the efficiency of the sintering process. It was reported that linear shrinkage of 5–20% is a measure of highly sintered materials^29,30^. Linear shrinkage, % in diameter for the sintered A/AT composites with different rutile content (0–40 wt%) is declared in Fig. 4. Noticeably, the linear shrinkage behavior of the sintered composites is directly proportional to the bulk density. For a small amount of rutile addition (10%), the bulk density was significantly enhanced, which resulted in increasing the the linear shrinkage. This can be explained by the grain boundary modification during the liquid phase sintering, as explained before. In contrast, the relatively small decrease in the density and linear shrinkage for higher levels of rutile may be attributed to the formed phases in each sample according to the XRD data, Fig. 2. Introducing more than 10% of rutile results in formation of more aluminium titanate, which has lower density and higher grain growth phenomena. As a conclusion, it is suggested that this reduction of linear shrinkage can be probably related to the grain growth of aluminium titanate and the formation of microcracks. The highest shrinkage rate of 21.64% is achieved by the 10 R composite, while the lowest one of 16.52% is given by the 0 R composite. This outstanding contraction rate achieved by adding only 10% rutile to alumina ceramics confirms the unique behavior of rutile addition.

### Microstructure examination

**Figure 5:**
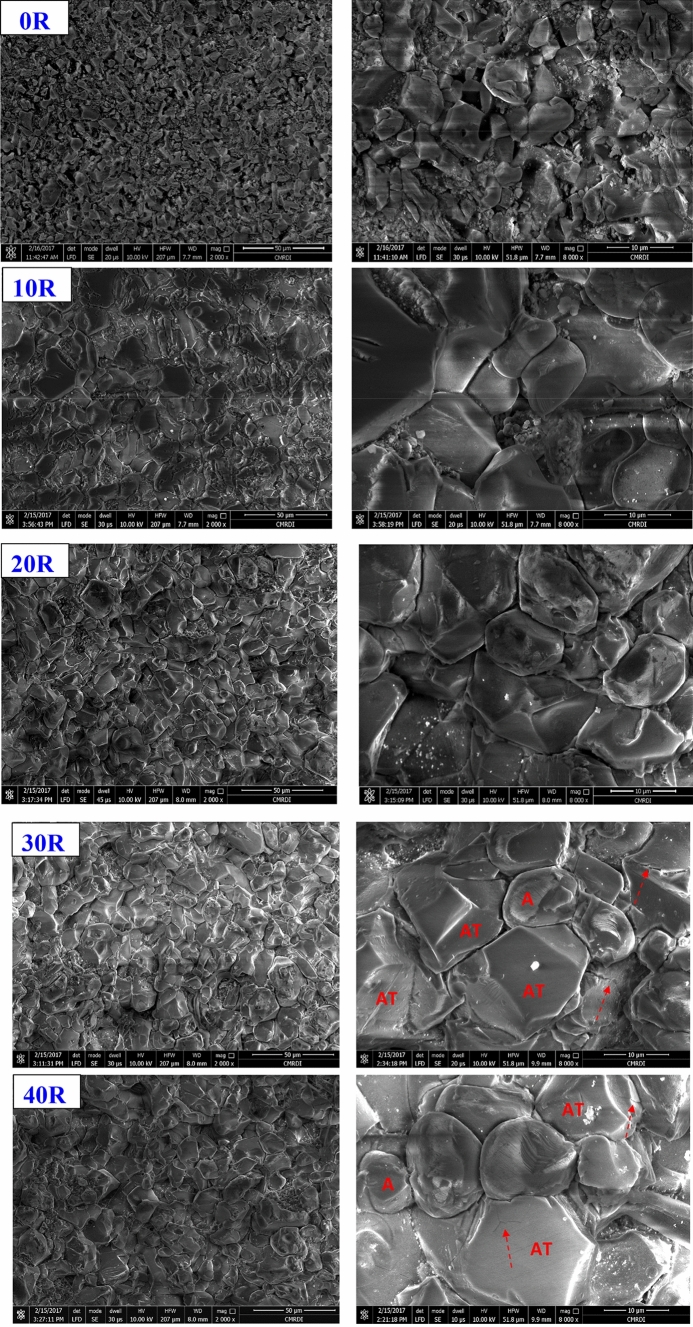
FE-SEM micrographs of the sintered A/AT composites with different rutile content (0–40 wt%).

FE-SEM micrographs with high and low magnifications of the different sintered A/AT composites are shown in Fig. 5. Microstructure of the different samples shows the intimate diffusion and distribution of the formed aluminum titanate with alumina structure that resulting from the homogenous mixing of the starting materials and the complete attained solid solution reaction. Reactivity of rutile clean source could be also taken into consideration. Concerning composite without rutile addition (0 R), alumina grains are distributed in various forms and sizes. They are distinctly present in equiaxed and cubic forms. Besides, some of them are as small as 2–5 µm. Other smaller grains are distributed in some areas of the alumina matrix. Moreover, some large grains not exceeding 10 µm are observed. The distribution of small and large grains throughout the matrix indicates a partial grain growth of alumina. The microstructure also showed some intergranular and open pores, which may be clarified by the partial grain growth of alumina. The presence of these pores results in the uncompacted structure between the grains, which explains the low density and high porosity of this sample as discussed before in the densification sections.

**Figure 6:**
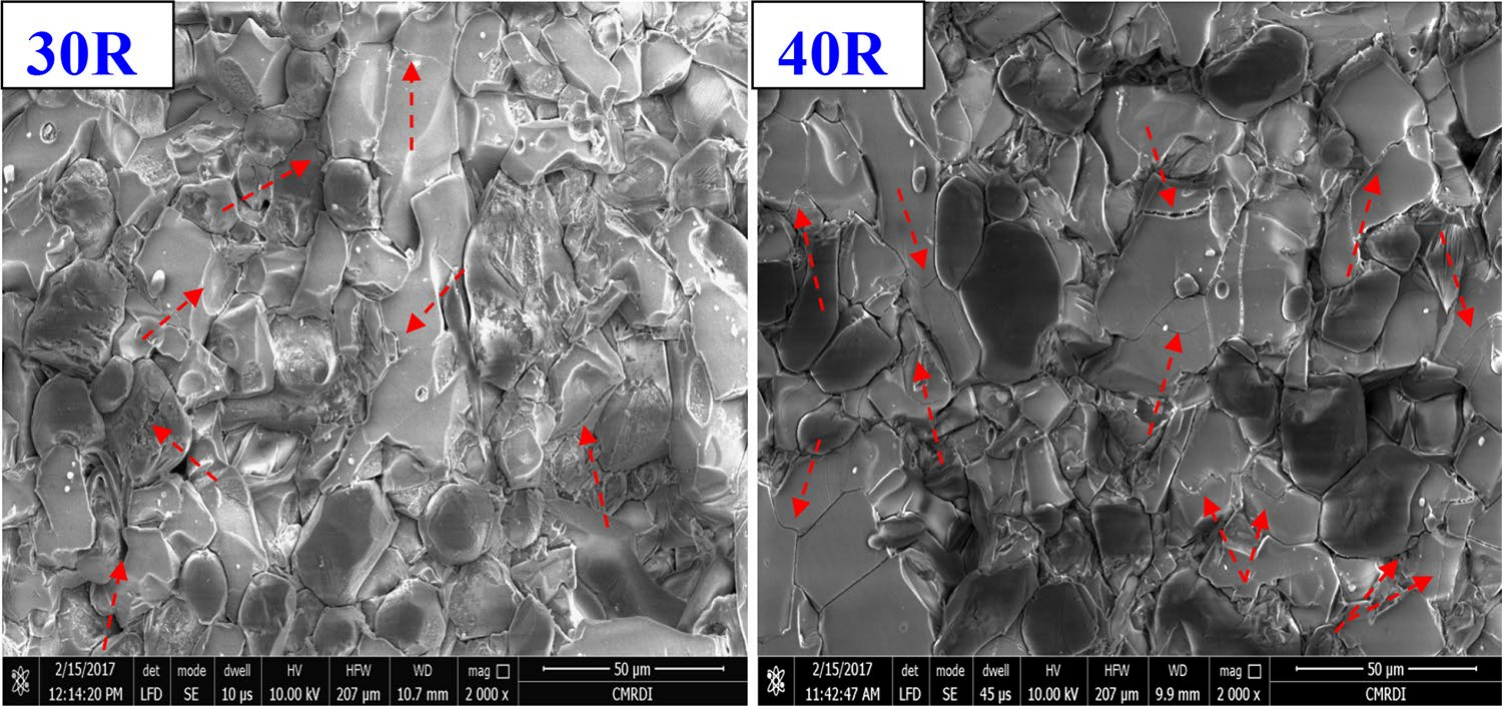
Microcracks distribution in sintered composites with high rutile content (30 and 40 wt%).

**Figure 7:**
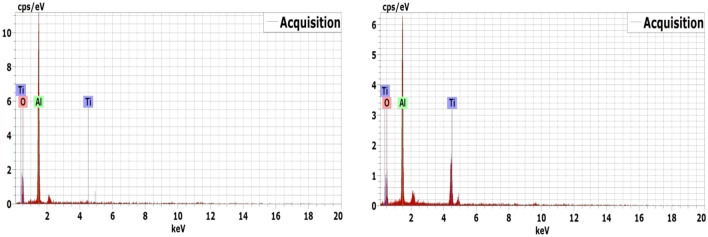
EDS analysis of alumina and aluminum titanate in the sintered 30 R and 40 R composites.

On the other hand, with the addition of 10 wt% rutile to alumina (10 R sample), some relatively larger grains have appeared, confirming the nucleation and formation of the AT structure. It is well known that AT grains have larger sizes than alumina ceramics. In addition, open pores have almost disappeared, and some closed pores are observed instead. The microstructure of the 10 R composite displayed reasonably dense and uniformly distributed grains in the order of 7–18 µm along with the very small grains of alumina. However, those in higher rutile added composites, such as 30 R and 40 R, seem to display a slightly greater grain size of about 10–20 µm. Moreover, some formed rounded and elongated grains are observed in these high rutile composites along with the equiaxed grains. The enhanced sintering kinetics for rutile added composites are related to the formation of a liquid phase and the second phase of AT, which fill the pores and enhance the densification^10,11,31^. Moreover, the presence of alumina with AT is expected to control the grain growth of both AT and alumina^8^. It is remarkable that with 10 wt% rutile addition, the size of alumina grains was almost like those in composite without rutile addition (0 R). In contrast, increasing rutile content higher than 20 wt% and up to 40 wt% led to abnormal grains growth of AT and finally to microcracking. This behavior can be attributed to that increasing rutile content leads to more formation of AT^6,10,11^. Consequently, the amount of existed alumina became small and not enough to completely prevent this grain growth behavior. So that, some large grains are observed in the composites with high rutile content addition (30 and 40 wt%). Also, some small microcracks are observed in the high magnification micrographs as illustrated in Fig. 6. The distribution and diffusion of alumina and AT grains (indicated with A and AT symbols) was confirmed by EDS analysis, Fig. 7.

### Mechanical properties

**Figure 8:**
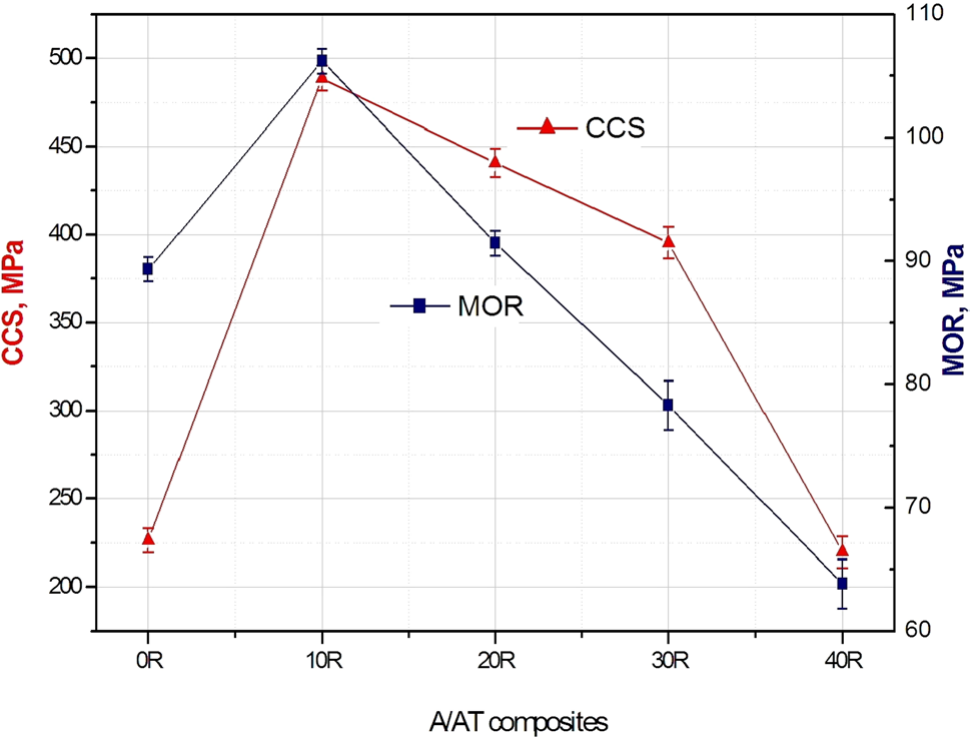
CCS and MOR of the sintered A/AT composites with different rutile content (0–40 wt%).

Mechanical properties in terms of flexural strength (modulus of rupture: MOR) and cold crushing strength (CCS) of the sintered A/AT composites measured at room temperature are demonstrated in Fig. 8. Noticeably, the correlation between mechanical strength and rutile addition showed the same trend of the densification parameters/rutile addition curve, Fig. 3. This indicates the strong relationship between strength and density. CCS and MOR values of 226.5 MPa and 89.3 MPa respectively are obtained for the 0 R sample. With adding 10 wt % rutile, CCS and MOR values are distinguishably improved to 488.7 MPa and 106.1 MPa, respectively. Afterward, their values are gradually reduced with the continual increase of rutile content (> 10 wt%) until achieved CCS and MOR values of 219.8 MPa and 63.8 MPa, respectively for 40 R composite. Increasing the mechanical strength for composite with 10 wt% rutile may be explained by reducing the number or size of pores during the bonding of alumina and AT grains via the formed liquid phase and solid solution reaction^10,11^. While the reduction in the mechanical strength values associated with the excessive addition of rutile (20–40 wt%) may be owing to the formed phases in each batch according to the XRD data in Fig. 2. At lower levels of rutile, the main phase was Al_2_O_3_. However, insertion of more rutile, makes Al_2_TiO_5_ the major phase. In which Al_2_O_3_ is denser and has higher mechanical strength. Additionally, this behavior can be attributed to the abnormal grains growth of AT grains attached to increasing its content and the higher porosity levels of these composites. This in turn led to initiation of microcracks formation, which increases with further addition of rutile (as shown in Figs. 5 and 6) and subsequently result in lowering the mechanical strength. Moreover, it can be noticed that the mechanical strength of the obtained samples in this work is significantly greater than those reported in the literature for AT synthesized from pure oxides with further modification by various additives employing several preparation methods (see Table 3 for some comparison).

**Table 3:** Mechanical and thermal properties of AT-based ceramic composites.

Material	Flexural strength (MPa)	Coefficient of thermal expansion (*10^–6^ K^−1^)	References
AT	8.53	1.09	^36^
AT-clay	8.61	0.94	^34^
AT-Talc	25.89	0.4	^34^
AT-MgO	38.4	–	^13^
AT-Talc	48.5	–	^13^
AT-Talc-feldspar	34.7	–	^13^
AT-mullite	37.5	–	^13^
AT-ZrTiO_4_	22	–	^31^
AT	–	0	^37^
AT-ZrSiO_4_	–	2.7	^37^
AT	30.84	− 0.927	^10^
Magnesium-AT	9.1	− 3.32	^11^
AT-Al_2_O_3_	63.8–106.1	− 2.34 to − 8.52	Our work

### Thermal expansion and its coefficient (LTE and CTE)

**Figure 9:**
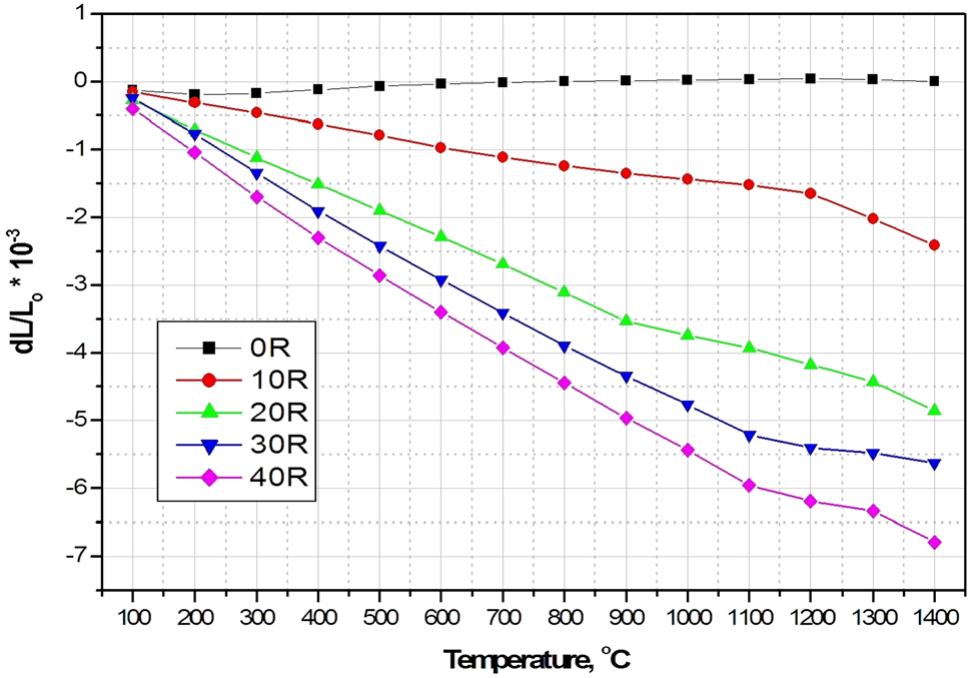
LTE of the different A/ AT composites as a function of temperature.

**Figure 10:**
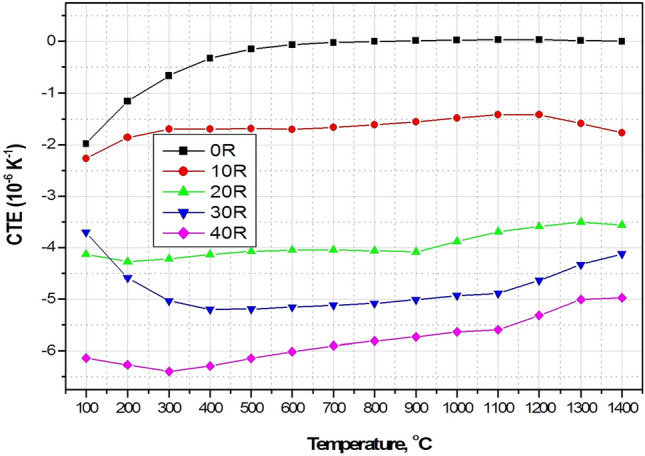
CTE of the different A/ AT composites as a function of temperature.

In various industrial sectors requiring fabrication and processing of high-temperature ceramic materials, many of these materials are deteriorated by the prompt changes of temperature that evolve large thermal stresses. Consequently, usability and productivity are limited. Therefore, very low thermal expansion materials are mandatory for severe thermal shock applications. Investigation of the thermal expansion behavior is very important in determining the material lifetime, suitability, and sustainability in high-temperature environments^29,30^. Linear thermal expansion (LTE) and its coefficient (CTE) of the sintered A/ AT composites (0–40 wt% rutile addition) as a function of temperature (from 100 to 1400 °C) are shown in Figs. 9 and 10. It was found that the thermal expansion behavior of 0 R composite shows a distinct attitude compared to the other rutile added composites. Comparison between the thermal expansion curves of rutile added and the non added composites revealed that a continuous decrease of the thermal expansion value as the rutile content increases (up to 40 wt %) was detected. As can be clearly seen, with further addition of rutile, the thermal expanstion values shift towards the higher negative values. According to XRD and microstructure analysis, this behavior is believed to be due to the continuous increase of aluminium titanate besides its grain growth. Crystals of AT are made of domains with various directions. As a result, the thermal expansion ratio of AT is anisotropic. Thus, amid the cooling process, a single crystal domain of the AT contracts resulting in the anisotropy and the evolution of microcracks. Otherwise, an expansion of the singular crystal domain of AT occurs amid the heating operation. Nevertheless, the expansion of the crystal domains was prohibited by the existence of microcracks^32,33^. Therefore, apparent expansion was not observed during heating of rutile added samples, which contain AT. This behavior is in good agreement with previous works^10,11,31,32,34–37^. It was found that 0 R sample gave the highest CTE_(100–1400 °C)_ value of 0.061 * 10^–6^ K^−1^ followed by 10 R composite with a marginally lower CTE _(100–1400 °C)_ value of -2.34* 10^–6^ K^−1^ and the lowest value of − 8.52 * 10^–6^ K^−1^ was recorded by 40 R composite. So that, addition of rutile to alumina was found to have a special and unparalleled role in decreasing its thermal expansion and hence enhancing its thermal performance. In addition, from Table 3 it can be seen that the coefficient of thermal expansion of our prepared AT- based composites are Cleary comparable with other AT- based materials synthesized from different starting materials and processing routes.

**Figure 11:**
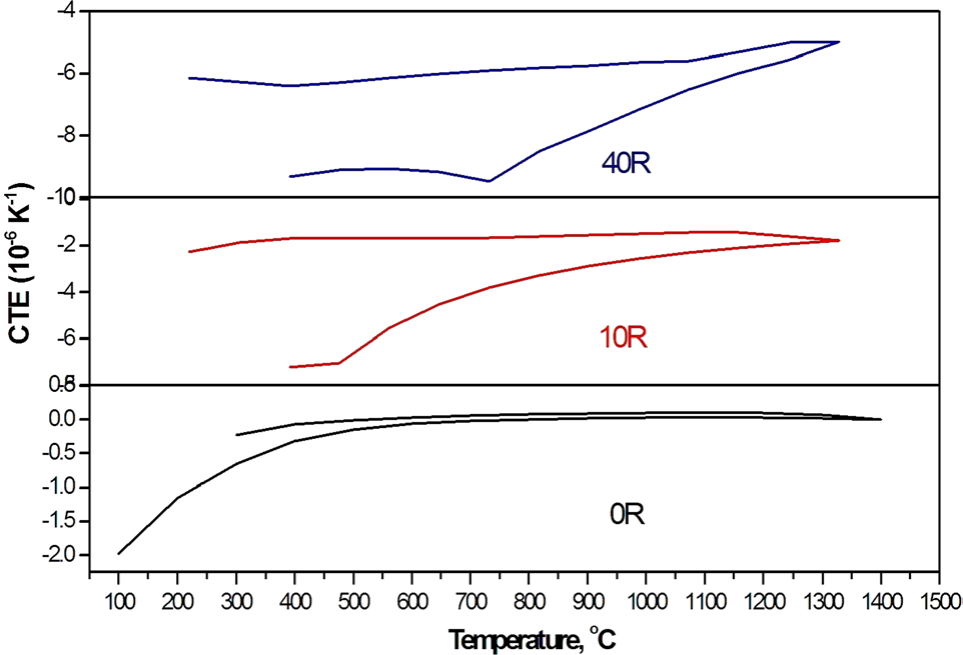
Heating/cooling curve of some selected A/ AT composites show the gradual change in the size of the formed hysteresis loop.

Moreover, cooling curves also behaved differently with all rutile added composites as shown in Fig. 11. Sintered composites exhibit a pronounced hysteresis loop, characteristic of composite materials having different thermal expansion coefficients. At higher levels of rutile addition, the hysteresis loop is quite larger, whereas the turning point of the expansion in cooling curves is continuously shifted towards the higher temperatures (from about 400 to about 700 °C) as rutile addition increases. The increased microcracks density was proved to be associated with the formation of more AT^12^. Indeed, the size of the hysteresis loop is significantly depending on the microstructure and the cracks volume. The grain size also plays an important role. The temperatures at which microcracks reopen are represented by the turning points in the cooling curves^13^. Beneath these temperatures, the amount of expansion was correlated with the density of the existing microcracks in the cooled samples. Otherwise, the expansion behavior during heating is controlled by these microcracks^13^. This can explain the small expansion starting at 400 °C on the cooling curve for the 10 R sample containing the lowest amount of AT and exhibits the lowest grain size among rutile added samples. It was also observed that this expansion increases with the increase of AT phase in the composite associated with the higher additions of rutile.

On the contrary, single-phase alumina sample (0 R) does not show such hysteresis area, Fig. 11. The absence of microcracks in this composite is the reason for this behavior^10,11^. In addition, this sample has the smallest grain size of all.

In conclusion, the addition of rutile to alumina, which resulted in the formation of AT along with alumina in the different A/AT composites, was found to be an effective way of enhancing the thermal performance of alumina. This was achieved by decreasing the thermal expansion, which in turn enhances the thermal shock resistance of the material. Moreover, the obtained composites show thermal stability without any decomposition during heating from 100 up to 1400 °C.

## Conclusions

In an attempt to develop a new high temperature and advanced material to keep up with industrial requirements, various Al_2_O_3_/Al_2_TiO_5_ composites were developed with high thermal and mechanical performance from a natural clean source and low cost. The proposed composites were produced from the solid solution reaction of calcined alumina and for the first time, from rutile ore extracted from the Egyptian black sand at a temperature of 1650 °C/2 h. Rutile was added to alumina with different content (0–40 wt%) to promote its sinterability and thermo-mechanical response. Highly dense composites with harmonic microstructural and enhanced mechanical strength were attained by increasing the rutile content. Composite with 10 wt% rutile gave the highest density of 3.6 g/cm^3^ and the highest CCS and MOR values of 488.73 MPa and 106.19 MPa, respectively. Notably, the addition of rutile has a substantial effect on promoting the thermal properties and thermal stability of the obtained composites up to the high-temperature of 1400 °C. This was achieved by decreasing their thermal expansion values, which in turn enhances their thermal shock resistance. It can be concluded that using a clean natural source such as rutile ore which already contains some stabilizers as Fe_2_O_3_, Al_2_O_3_, SiO_2_, ZrO_2_, and MgO has played a significant rule in lowering the cost of the preparation process and also in enhancing the properties of the prepared composites. This was the main motivation behind this work, to encourage using clean sources, instead of higher cost purified starting materials. Thus, sintered Al_2_O_3_/ Al_2_TiO_5_ composites can be considered as a promising high temperature material for advanced structural and thermal applications.
